# Longitudinal Analysis of the Temporal Evolution of *Acinetobacter baumannii* Strains in Ohio, USA, by Using Rapid Automated Typing Methods

**DOI:** 10.1371/journal.pone.0033443

**Published:** 2012-04-12

**Authors:** Brooke K. Decker, Federico Perez, Andrea M. Hujer, Kristine M. Hujer, Geraldine S. Hall, Michael R. Jacobs, Wondwossen A. Gebreyes, Scott T. Zoll, Christian Massire, Mark W. Eshoo, David J. Ecker, Philip N. Rather, Robert A. Bonomo

**Affiliations:** 1 Department of Medicine, Case Western Reserve University School of Medicine, Cleveland, Ohio, United States of America; 2 Research Service, Louis Stokes Cleveland Department of Veterans Affairs Medical Center, Cleveland, Ohio, United States of America; 3 Cleveland Clinic Pathology and Laboratory Medicine Institute, Cleveland, Ohio, United States of America; 4 Department of Pathology, Case Western Reserve University School of Medicine, Cleveland, Ohio, United States of America; 5 College of Veterinary Medicine, Ohio State University, Columbus, Ohio, United States of America; 6 Ibis Biosciences Inc., Abbott Molecular, Inc., Carlsbad, California, United States of America; 7 Research Service, Veterans Affairs Medical Center, Decatur, Georgia, United States of America; 8 Department of Microbiology & Immunology, Emory University School of Medicine, Atlanta, Georgia, United States of America; 9 Department of Molecular Biology and Microbiology, Case Western Reserve University School of Medicine, Cleveland, Ohio, United States of America; 10 Department of Pharmacology, Case Western Reserve University School of Medicine, Cleveland, Ohio, United States of America; Los Angeles Biomedical Research Institute, United States of America

## Abstract

Genotyping methods are essential to understand the transmission dynamics of *Acinetobacter baumannii*. We examined the representative genotypes of *A. baumannii* at different time periods in select locations in Ohio, using two rapid automated typing methods: PCR coupled with electrospray ionization mass spectrometry (PCR/ESI-MS), a form of multi-locus sequence typing (MLST), and repetitive-sequence-based-PCR (rep-PCR). Our analysis included 122 isolates from 4 referral hospital systems, in 2 urban areas of Ohio. These isolates were associated with outbreaks at 3 different time periods (1996, 2000 and 2005–2007). Type assignments of PCR/ESI-MS and rep-PCR were compared to each other and to worldwide (WW) clone types. The discriminatory power of each method was determined using the Simpson's index of diversity (*DI*). We observed that PCR/ESI-MS sequence type (ST) 14, corresponding to WW clone 3, predominated in 1996, whereas ST 12 and 14 co-existed in the intermediate period (2000) and ST 10 and 12, belonging to WW clone 2, predominated more recently in 2007. The shift from WW clone 3 to WW clone 2 was accompanied by an increase in carbapenem resistance. The *DI* was approximately 0.74 for PCR/ESI-MS, 0.88 for rep-PCR and 0.90 for the combination of both typing methods. We conclude that combining rapid automated typing methods such as PCR/ESI-MS and rep-PCR serves to optimally characterize the regional molecular epidemiology of *A. baumannii*. Our data also sheds light on the changing sequence types in an 11 year period in Northeast Ohio.

## Introduction

*Acinetobacter baumannii* has emerged worldwide as a cause of infection among seriously ill patients [1]. *A. baumannii* is a frequent cause of outbreaks in hospitals and long term care facilities, where this pathogen is associated with prolonged hospitalizations and possibly increased mortality [2], [3]. Also, military medical facilities treating personnel serving in Iraq and Afghanistan have experienced *A. baumannii* outbreaks [4], [5], [6]. The remarkable ability of *A. baumannii* to display resistance to multiple classes of antibiotics, including carbapenems, poses a serious therapeutic challenge and likely contributes to its global success as a healthcare-associated pathogen [1], [7], [8].

Genetic typing is an essential tool in understanding the transmission dynamics and temporal evolution of *A. baumannii*. For instance, typing of DNA digests using pulsed field gel electrophoresis (PFGE) enhances the epidemiological investigation of outbreaks by demonstrating highly related or indistinguishable isolates, suggesting transmission from a common source or from patient-to-patient [9], [10]. Comparing PFGE typing of *A. baumannii* from different institutions requires the careful standardization of protocols [11]. In this regard, the combination of PFGE, ribotyping, and amplified fragment length polymorphism (AFLP) permitted the identification of important international clones of *A. baumannii* from different hospitals in Europe [12], [13], [14]. These strains, European clones 1–3, are also found globally and thus are now termed worldwide (WW) clones [12]. Multi-locus sequence typing (MLST), a method suitable for pathogens with wide genomic, temporal and spatial variations, has permitted further characterization of the population structure, genetic diversity and distinctness of the WW clones of *A. baumannii* [15]. The cost and time-consuming nature of MLST, although becoming more accessible, limit its current applicability except in specialized circumstances [16].

Rapid and automated typing methods are increasingly applied to *A. baumannii*. PCR coupled with electrospray ionization mass spectrometry (PCR/ESI-MS) analyzes the base-composition of amplicons from six housekeeping genes, generating a unique signature that corresponds to a sequence type (ST) in a database [17], [18]. These genes, however, differ from the seven housekeeping genes amplified and sequenced to perform MLST. Consequently, the STs generated by PCR/ESI-MS do not correspond to those of MLST schemes. Automated PCR/ESI-MS has been used to investigate *A. baumannii* from civilian and military treatment facilities revealing sequence types corresponding to the WW clones [3], [6], [17],[19]. Automated repetitive-sequence-based-PCR (rep-PCR) has also been employed to type *A. baumannii*. Using rep-PCR, a unique profile of bands is generated by the resolution of amplified DNA fragments in a gel matrix. Automated rep-PCR has been applied to the analysis of local outbreaks and to illustrate the global spread of carbapenem-resistant lineages of *A. baumannii* [3], [12].

Insights into the molecular epidemiology of *A. baumannii* gained from rapid automated methods aid in surveillance, demonstrate the temporal pattern of strain replacement, and may lead to successful interventions to control this important pathogen. In this study, we investigate temporal changes in the molecular epidemiology of *A. baumannii* in two urban centers in Ohio, and explore the discriminatory ability of two rapid automated methods, PCR/ESI-MS and rep-PCR.

## Methods

### Ethics Statement

Expedited approval was obtained from the Institutional Review Board at Louis Stokes Cleveland VA Medical Center, IRB # 09084-H04. The bacterial isolates analyzed in this study belong to the microbiological collections of each hospital and were obtained as part of routine clinical care in the past. Furthermore, all patient identifiers had been previously removed and data were analyzed anonymously. Therefore, the Institutional Review Board waived the need to obtain written or verbal consent.

### Bacterial Isolates

**Figure 1:**
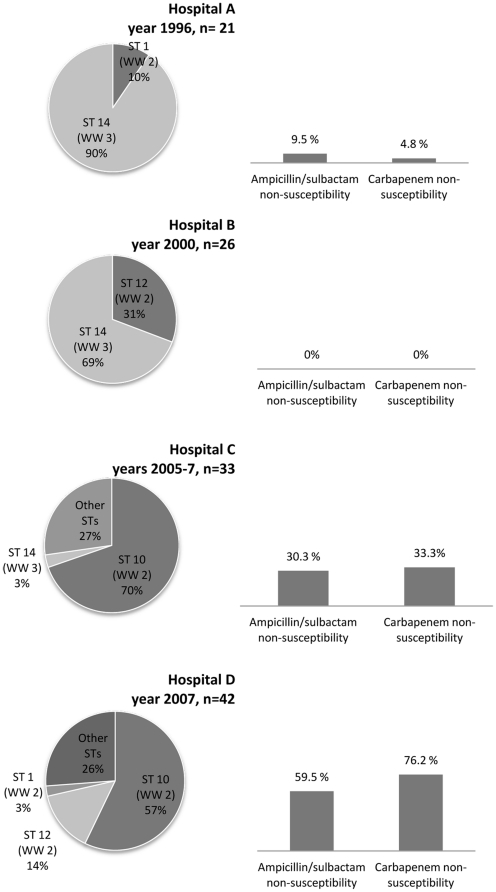
Origin, sequence type and antibiotic susceptibility of *Acinetobacter baumannii* from Ohio. Distribution of 122 *A. baumannii* isolates by hospital of origin (hospitals A, B, C and D), year, sequence type (ST) determined by PCR/ESI-MS, worldwide clone type (WW) and susceptibility to carbapenems and ampicillin/sulbactam.

A total of 122 single-patient isolates of *A. baumannii* were studied. The isolates were identified and antimicrobial susceptibility testing was performed with VITEK® (bioMérieux, Durham, NC) or Microscan (Siemens Healthcare, Deerfield, IL), at their respective hospitals of origin. These were four tertiary-care hospital systems from two cities in Ohio, which experienced outbreaks of *A. baumannii* at three different time periods: 1996, 2000 and 2005–2007 (Figure 1). Hospital systems A, B, and D are located in the Cleveland, Ohio regional area and hospital system C is located approximately 140 miles south in Columbus, Ohio. The isolates were obtained from microbiological cultures of various types of specimens (e.g., blood, sputum, urine, wounds) and belonged to different patient populations (i.e., ICU, non-ICU, adult, pediatric, medical, surgical, trauma and burn patients).

### PCR/ESI-MS

Overnight cultures were diluted 1∶50 in Tris-EDTA buffer and were boiled at 99°C for 15 minutes prior to use. PCR/ESI-MS was performed using the *Acinetobacter* genotyping kit on the T5000™ Biosensor (Ibis Biosciences, Inc., Abbott Molecular, Inc., Carlsbad, CA). The base composition analysis was completed using the provided software. The software compared our amplicons to previously obtained sequences stored in a database in order to assign the sequence type (ST) of each strain, as previously described and validated [17]. PCR/ESI-MS STs were correlated with WW clones 1–3, according to previously published studies [6], [17], [19].

### Rep-PCR

Genomic DNA was extracted from bacterial isolates using the UltraClean™ Microbial DNA Isolation Kit (MoBio Laboratories, Carlsbad, CA). PCR amplification was performed using the DiversiLab® (bioMérieux, Athens, GA) *Acinetobacter* fingerprinting kit, according to the manufacturer's instructions. Rep-PCR products were separated by electrophoresis on microfluidic chips and analyzed with the Agilent 2100 Bioanalyzer (Agilent Technologies, Santa Clara, CA). The resulting band patterns were compared in order to generate a dendrogram using two different statistical methods: Pearson correlation (PC) and the modified Kullback–Leibler (KL). Both methods calculate similarity using relative band intensity, however, PC is more “band intensity” based and KL is more “band presence” driven. As validated in previous studies, isolates having band patterns with ≥95% similarity were considered genetically related strains (genotypic clusters) [3], [12]. In a further analysis, isolates were grouped on the basis of ≥98% similarity, in order to detect dissemination of identical or near-identical strains.

### Discriminatory ability of PCR/ESI-MS and rep-PCR

Strain types obtained by PCR/ESI-MS and rep-PCR patterns were compared to establish concordance between the two typing methods. The discriminatory abilities of PCR/ESI-MS and rep-PCR were compared by contrasting the number of unique types determined by each method, and by determining the Simpson's index of diversity (*DI*). This is an index of discrimination for bacterial typing methods and was calculated using the following formula [20], [21]: 
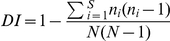
In the above equation, *N* is the total number of strains in the sample population, *S* is the total number of types described, and *n_i_* is the number of strains belonging to the *i* ^th^ type. *DI* ranges from 0 to 1; a value closer to 1 represents larger diversity. 95% confidence intervals were estimated for *DI*, according to previously described equations [22]. *DI* and 95% confidence intervals were calculated for PCR/ESI-MS, rep-PCR (analyzed with both the KL and PC methods) and for the combination of PCR/ESI-MS and rep-PCR.

## Results

The 122 *A. baumannii* isolates included in this study were represented by 17 different PCR/ESI-MS STs and by 29 and 27 different rep-PCR types, when analyzed with the KL and PC methods, respectively. Overall, ST 10 (n = 47), ST 14 (n = 38) and ST 12 (n = 14), were the predominant ST types established by PCR/ESI-MS (Table 1 and Figure 1).

**Table 1:** Predominant PCR/ESI-MS sequence types and rep-PCR types analyzed by Kullback-Leibler (KL) and Pearson correlation (PC) methods.

PCR/ESI-MS sequence type (ST)	No.	rep-PCR Kullback-Leibler (KL) type	No.	rep-PCR Pearson correlation (PC) type	No.
ST 10*	47	KL 1	16	PC 1	10
ST 12*	14	KL 2	23	PC 2	20
ST 1*	3	KL 3	10	PC 3	5
ST 86*	1	KL 4	7	PC 4	21
		KL 5	5	PC 5	5
		Unique	4	PC 6	2
				Unique	2
ST 14	38	KL 5	28	PC 7	28
		KL 6	7	PC 8	7
		Unique	3	Unique	3
ST 54	4	KL 7	2	PC 9	3
		Unique	2	Unique	1

* rep-PCR did not differentiate among ST 10, ST 12, ST 1 and ST 86.

### Change of predominant sequence types over time

**Figure 2:**
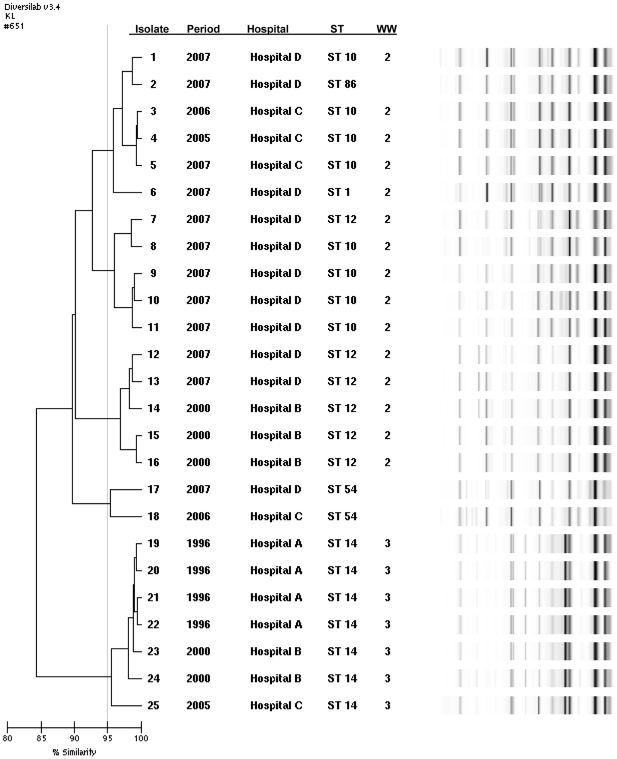
Genetic similarity among *A. baumannii* isolates. Representative *A. baumannii* isolates typed by rep-PCR, analyzed with the Kullback-Leibler method. Five strain types with >95% similarity are illustrated, and further discriminated by year, hospital of origin, PCR-ESI/MS sequence type (ST), and worldwide (WW) clone types.

Our results indicate that different sequence types or separate “waves” of *A. baumannii* spread through different parts of Ohio in past decades. Figures 1 and 2 illustrate the sequence and rep-PCR types of *A. baumannii* from the four hospital systems in the three different time periods analyzed. Among the 21 isolates from the initial period of 1996, ST 14 was the predominant type (90% of isolates). ST 14 corresponds to WW clone 3. Isolates from 2000 were characterized by the continued predominance of ST 14 (69% of isolates). In the 2000 period however, 31% of isolates belonged to ST 12, corresponding to WW clone 2. Among isolates from the more recent period of 2007, ST 14 was not identified, while ST 12 represented only 8% of isolates. The predominant type in 2005–2007 was ST 10 (63%), which also belongs to WW clone 2 (Figures 1 and 2 and Table 1). Susceptibility to carbapenems and ampicillin/sulbactam was maintained in isolates from 1996 and 2000. More recent isolates, from 2005–2007, displayed increasing resistance to these agents: non-susceptibility to carbapenems was 76.2% among *A. baumannii* isolates from 2007 (Figure 1).

There were many instances of almost identical rep-PCR types (defined as ≥98% similar) present in different locations (data not shown): hospital A shared isolates that were 99.2% similar (belonging to ST 14) with hospital B, and a ST 1 isolate from hospital A was 98% similar to ST 10 isolates from hospital D. Strains belonging to ST 10 and ST 68 with ≥98% similarity were found in hospitals C and D, while several ST 10/ST 12 isolates from hospitals B and D were 98.1% similar.

### Comparison and discriminatory power of PCR/ESI-MS and rep-PCR

Isolates with the same STs belonged to multiple rep-PCR types (Table 1). For example, the 38 isolates identified as ST 14 were classified into five different rep-PCR types: two predominant types, accounting for 74% and 18% of isolates respectively, and 3 unique types.

The Simpson's index of diversity (*DI*) was determined to compare the discriminatory ability of PCR/ESI-MS and rep-PCR, analyzed by two different methods (KL and PC). Additionally, *DI* was calculated for the combined analysis of strain types with both PCR/ESI-MS and rep-PCR. Table 2 shows different *DI* values for PCR/ESI-MS and rep-PCR, with confidence intervals that do not overlap. The value of *DI* was larger for the combination of PCR/ESI-MS and rep-PCR, indicating a superior discriminatory power. There is, however, overlap of the confidence interval with that of rep-PCR alone indicating that differences in *DI* may not be significant. Note that both methods of rep-PCR analysis (KL and PC) have a very similar *DI* and confidence intervals, indicating essentially identical discriminatory abilities.

**Table 2:** Simpson's Diversity index (*DI*) of PCR/ESI-MS, rep-PCR and the combination of PCR/ESI-MS and rep-PCR.

Typing method	Number of different types	Percentage of isolates with the most frequent type	Simpson's *DI*	95% Confidence interval
PCR/ESI-MS	17	39	0.744	0.692–0.796
rep-PCR(Kullback-Leibler)	29	23	0.884	0.854–0.914
rep-PCR(Pearson correlation)	27	23	0.882	0.852–0.912
PCR/ESI-MS+rep-PCR (Kullback-Leibler)	34	23	0.899	0.869–0.929
PCR/ESI-MS+rep-PCR(Pearson correlation)	33	23	0.903	0.872–0.934

## Discussion

This decade-long study provided a unique view of the molecular epidemiology of *A. baumannii* in two urban areas of a Midwestern state in the USA. Of primary importance, we observed that there was a change in the predominant sequence types of *A. baumannii* associated with outbreaks from the four hospital systems, shifting from carbapenem-susceptible strains corresponding to WW clone 3 in 1996, to carbapenem-resistant strains corresponding to WW clone 2 in 2007. Secondly, we noted discordances in the type assignments and differences in the discriminatory abilities of two rapid automated typing methods. The higher discrimination of rep-PCR makes it useful to chart hospital outbreaks, whereas the PCR/ESI-MS delineates the underlying population structure. Combining both methods may provide a more accurate differentiation of isolates when performing molecular epidemiology studies comprising isolates of different years obtained from several hospitals.

The isolates of *A. baumannii* analyzed in this study were restricted to outbreak strains rather than those collected prospectively and continuously. Nevertheless, we found, considerable diversity, 17 to 29 strain types, depending on the typing method used. Although our study was limited to isolates from four hospital systems and three different time periods, we believe that our data provide early clues as to the regional transmission dynamics of *A. baumannii* in Ohio and perhaps even on a global level. The initial description of European clones 1–3 dates from the mid-1990s and was inferred from ribotyping, AFLP and PFGE typing of *A. baumannii* strains associated with outbreaks throughout that continent [15], [23], [24], [25], [26]. Presently, these clone types of *A. baumannii* extend beyond Europe. For example, clonal clusters corresponding to what is now termed worldwide (WW) clone 2 are widely disseminated in multiple Chinese cities, and WW clone 2 has been the predominant clonal type in a medical center in Australia for more than a decade [27], [28]. A contemporary international survey of *A. baumannii* non-susceptible to carbapenems typed with rep-PCR, PFGE and multiplex PCR, which led to change the designation of European clones to WW clones, confirmed the universal presence of clones 1–3, and identified clone 2 as the largest and most widespread type [12]. Our analysis identified an international strain of *A. baumannii*, WW clone 3, in the Midwestern USA as early as 1996. This surprising result resonates with ongoing questions as to the meaning and origin of these successful global strains [29].

Previously, identification of WW clones 1–3 in the USA occurred among *A. baumannii* obtained from patients at the Walter Reed Army Medical Center in 2003–2005, and was subsequently confirmed in isolates from 2006–2007 [6], [19]. In these studies, genetic typing with PCR/ESI-MS determined that WW clones 1–3 were represented. Similarly, contemporary *A. baumannii* from the National Naval Medical Center also included AFLP types belonging to WW clones 1–3 [30]. This is in contrast with isolates obtained in 2005–2007 and in 2008–2009 from civilian hospitals across the United States (California, Arizona, Kentucky, Illlinois, Pennsylvania, New York, Florida, Missouri and Nevada), where WW clone 2 predominated and WW clones 1 and 3 were rarely found [19], [31]. Surveys of isolates recovered in 2001–2004 from 17 European countries revealed the co-dominance of WW clones 1 and 2, to the exclusion of WW clone 3 [32]. The emergence in Italy and Greece of WW clone 2 among isolates obtained between 2005 and 2009 also supports this shift. [33], [34]. Our data confirms the replacement of WW clone 3 and the predominance of strains related to WW clone 2 among *A. baumannii* causing outbreaks in civilian hospitals in Ohio.

The determination of WW clones of *A. baumannii* has proven to be consistent, even when diverse typing methods, such as rep-PCR and PCR/ESI-MS, are used [12], [17]. For instance, isolates classified by PCR/ESI-MS as ST 1, ST 10, and ST 86 (all belonging to WW clone 2) shared the same rep-PCR type. Of note, the base composition of ST 1, ST 12, and ST 86 are single locus variants of ST 10 in the PCR/ESI-MS scheme: they only differ in one of the 6 housekeeping genes examined. Nevertheless, disagreement in type assignment, for instance between PFGE and PCR/ESI-MS or rep-PCR, can also occur [17]. In this study, there was a trend towards a higher discriminatory ability using both PCR/ESI-MS and rep-PCR, suggesting a significant benefit in combining these two typing methods for *A. baumannii*. The value of complementary typing methods has been previously recognized for *Aspergillus* and *Staphylococcus* [14], [35], [36], [37]. Our data also demonstrate that the two different methods of analysis for rep-PCR (KL and PC) result in very similar discriminatory abilities (Table 2), but separate isolates into slightly different groupings (Table 1). The presence of almost identical rep-PCR types in different hospitals, even after increasing the threshold of similarity from ≥95% to ≥98%, suggests potential clonal dissemination of *A. baumannii*. This data must be interpreted carefully, however, since we cannot provide other epidemiological evidence of patient-to-patient spread occurring between hospitals.

The factors behind the widespread distribution of WW clones of *A. baumannii* are not fully elucidated. Resistance to carbapenems may in part explain the success of WW clone 2 in Ohio (and globally), but we must keep in mind that the understanding of antibiotic resistance and clonality in *A. baumannii* is evolving [38]. Commonly, up regulation of the intrinsic β-lactamase gene *bla*_OXA-51-like_ is considered the most prevalent mechanism of carbapenem resistance among *A. baumannii*, whereas the *bla*_OXA-23-like_ gene is the predominant acquired mechanism of carbapenem resistance, followed by the presence of *bla*_OXA-58-like_ and *bla*_OXA-24/40-like_ genes [12]. Nevertheless, isolates belonging to WW clone 2 are frequently susceptible to carbapenems [25], [27], [28]. This is underscored by our analysis, where WW clone 2 isolates from 2000 were susceptible to carbapenems. Furthermore, in the years 2005–2007, there was coexistence of WW clone 2 isolates resistant and susceptible to carbapenems (Figure 1). Two previously published, larger analyses of the carbapenem-resistant isolates included in this study revealed *bla*_OXA-23_ and *bla*_OXA-24/40_ in approximately 45% and 10% of strains from hospital D, respectively [3]. In hospital C only 13% of isolates harbored *bla*_OXA-23_, but 80% of isolates had IS*AbaI* linked to *bla*_OXA-66_, a *bla*_OXA-51_-like gene [39].

In conclusion, an approach combining two different typing methods increases our understanding of the molecular epidemiology of *A. baumannii* in Ohio, revealing the regional predominance, temporal evolution, and progression of certain strain types. Furthermore, our observations also help illustrate the potential utility of combining rapid automated typing methods, such as PCR/ESI-MS and rep-PCR, in the study of *A. baumannii*. More importantly, we also show that WW clone 3 was present in Ohio at almost the same time as it was described in Europe. Our data also demonstrates the replacement of WW clone 3 with WW clone 2 as the predominant clone type, a pattern that is similar to national and international trends. Despite limitations that challenge our understanding of the transmission dynamics of *A. baumannii*, rapid and automated molecular typing methods such as PCR/ESI-MS and rep-PCR have demonstrated significant utility in evaluating and documenting the dissemination of *A. baumannii* in various geographic locations and time periods.
